# A Prototype Framework Design for Assisting the Detection of Atrial Fibrillation Using a Generic Low-Cost Biomedical Sensor

**DOI:** 10.3390/s20030896

**Published:** 2020-02-07

**Authors:** Jesús Pérez-Valero, Antonio-Javier Garcia-Sanchez, Manuel Ruiz Marín, Joan Garcia-Haro

**Affiliations:** 1 Department of Information and Communication Technologies, Universidad Politécnica de Cartagena (UPCT), Campus Muralla del Mar, E-30202 Cartagena, Spain; jesus.perez@edu.upct.es (J.P.-V.); joang.haro@upct.es (J.G.-H.); 2 Department of Quantitative Methods, Law and Modern Languages, Universidad Politécnica de Cartagena (UPCT), E-30202 Cartagena, Spain; manuel.ruiz@upct.es

**Keywords:** biomedical sensor, RR interval, R peak, ECG, logistic regression

## Abstract

Cardiovascular diseases are the leading cause of death around the world. As a result, low-cost biomedical sensors have been gaining importance in business and research over the last few decades. Their main benefits include their small size, light weight, portability and low power consumption. Despite these advantages, they are not generally used for clinical monitoring mainly because of their low accuracy in data acquisition. In this emerging technological context, this paper contributes by discussing a methodology to help practitioners build a prototype framework based on a low-cost commercial sensor. The resulting application consists of four modules; namely, a digitalization module whose input is an electrocardiograph signal in portable document format (PDF) or joint photographic expert group format (JPEG), a module to further process and filter the digitalized signal, a selectable data calibration module and, finally, a module implementing a classification algorithm to distinguish between individuals with normal sinus rhythms and those with atrial fibrillation. This last module employs our recently published symbolic recurrence quantification analysis (SRQA) algorithm on a time series of RR intervals. Moreover, we show that the algorithm applies to any biomedical low-cost sensor, achieving good results without requiring any calibration of the raw data acquired. In addition, it has been validated with a well-accepted public electrocardiograph (ECG) data base, obtaining 87.65%, 91.84%, and 91.31% in terms of sensitivity, specificity and accuracy, respectively.

## 1. Introduction

Over the last few decades, life expectancy has increased considerably due to better quality in living standards and constant progress in medicine, among other causes [1]. The consequential increase in population has significant social impact in terms of healthcare requirements. In this regard, healthcare cost containment is a crucial factor which implies strict control of medicines, medical equipment, and hospital care expenditures [2]. There is, thus, a need to develop and implement groundbreaking devices able to provide medical services at an affordable cost. This is particularly true for populations with limited access to basic healthcare services.

Cardiovascular diseases (CVDs) are the leading cause of death in the world [3]. In 2016, over 17.9 million people died of CVDs, representing 31% of all global deaths by disease. Of these deaths, 85% are due to heart attacks and strokes, and about three quarters take place in countries with low to middle average incomes [4].

To address these troubling statistics, a possible approach consists of thoroughly studying the functioning of patients’ hearts using portable devices. They can gather an enormous amount of data through recurrent heart tests which can be carried out at home, while patients sleep or go about their daily activities. So, monitoring patients with portable sensors is a way to improve life quality; especially for elder individuals and people with mobility problems, or to extend access to a health protection system. This type of monitoring provides an efficient and cost-effective alternative to on-site clinical monitoring [5]. Systems equipped with low-cost portable sensors are useful and feasible diagnostic tools for the real-time monitoring of important physiological indicators in such domestic/external facilities [6]. In the same context, wearable sensors are gaining popularity and attracting the attention of many researchers and technological companies [7]. Consequently, many leading corporations such as Apple and Samsung are spending large amounts of money on developing wearable devices focused on heart fitness. The set of features integrated in a single wrist-worn device will provide relevant information about the individual in almost real time. The main drawback to this approach is its low accuracy in ECG data acquisition. Interestingly, our methodology does not require ECG data calibration to provide good results.

The problem of data quality in ECG sensors has also been tackled in [8], where authors developed a single-chip based wearable ECG hardware system for monitoring patients and offer a high-quality ECG signal by applying three types of filters. Nevertheless, they did not focus on a heart disease classification approach. The authors of Reference [9] proposed a multi-sensor data fusion scenario in order to improve the quality of heart disease detection. They used a kernel random forest ensemble with several time and frequency domain features for their classification method. Even though they attained 98% accuracy working on a continuous stream of data, they incur in network bandwidth and battery consumption. Contrastingly, our design is single-device oriented, uses only RR intervals (time between two consecutive heartbeats) as input, and requires no network configuration.

Parallel to the development of these portable sensors for health monitoring, a large set of algorithms has been implemented to understand, detect, and classify different diseases. For instance, during the last decade, the design of different algorithms for the analysis of electrocardiogram signals has attracted the interest of many researchers. The detection and classification of different heart arrhythmias, in particular atrial fibrillation (AF), has played a central role since AF is a leading preventable cause of recurrent stroke for which early detection and treatment are critical.

Some relevant algorithms for the detection of AF are the ones based on the analysis of the P-wave [10,11], or those that utilize a large set of features obtained from ECGs in an artificial neural network [12,13,14] or in a deep learning approach [15,16]. Furthermore, it is worth mentioning that several recent clinical trials on large populations focusing on AF detection have been carried out [17,18,19]. In those clinical studies ECG data were obtained with portable sensors achieving outstanding results.

However, some limitations of these algorithms are: the high dependence on the robustness of the training data, the requirement for a filter preprocessing phase, and the fact that they are built upon a large set of features that are usually computationally complex to analyze, among others.

In order to overcome some of these limitations, several algorithms for the automatic detection of AF, based only on RR intervals (time between two consecutive heart beats), have been introduced [20,21,22].

Our work is aimed at developing a generic application and its associated methodology to be able to apply a predictive algorithm over the RR intervals acquired from any low cost biomedical sensor. In principle, this will not be easy to carry out due to the expected low precision of most of the portable sensors on the market. Portable sensors or wearables for this purpose, such as Apple Watch Series 4 [23], MySignals [24] or Qardio [25], provide electrocardiogram data in PDF format, which could be used as the input to our application. Regarding the low accuracy of these devices, it would be necessary to figure out a calibration procedure. In order to rapidly create a fully functional prototype, and taking low-cost and publicly available data into consideration, we use a commercially available portable electrocardiograph sensor called Kardia Mobile from AliveCor [26]. Albeit, Kardia Mobile has some limitations recently published [27,28] in comparison to the data accuracy of a 12-lead gold standard ECG recording. The MAC800 Healthcare [29] is employed as the 12-lead gold ground truth standard ECG equipment. For reproducibility purposes, the source code of the application developed, as well as its corresponding updates, can be found in Reference [30]. Furthermore, a possible MATLAB functional diagram is shown in Appendix A.

In addition, in this paper, we use our recently proposed predictive algorithm to detect atrial fibrillation, which is based on Symbolic Recurrence Quantification Analysis and can be found in Reference [31]. In order to maintain a self-contained paper, its more important features will also be summarized here. The use of Symbolic Recurrence Quantification Analysis (SRQA) [32] will provide us with a framework to analyze dynamic changes in a time series. This time series will be extracted from electrocardiogram (ECG) signals. More concretely, RR intervals are the measurement processed by our algorithm. The use of RR intervals as the unique input for our algorithm reduces the complexity of the approach and allows us to create a robust, simple and reliable solution for short and long term ECG monitoring. Specifically, we constructed a logistic regression algorithm that employs SRQA over RR interval time series to detect AF. The resulting approach can differentiate between normal sinus individuals and patients with AF. Furthermore, in order to show the power of our algorithm, we performed a logistic regression model that provides outstanding results in terms of power accuracy.

Our main contributions are: (i) the integration in an application of our computing time efficient algorithm; (ii) the fact that our algorithm only employs RR intervals as input so it can virtually operate with any low-cost sensor; and (iii) predictive power accuracy not being affected by a data calibration procedure.

The rest of this paper is organized as follows. Section 2 outlines the symbolic dynamics approach as well as the logistic model for the classification algorithm. Section 3 elaborates the methodology for applying the algorithm to generic low-cost devices. This section also presents the digitalization phases, the determination of RR intervals, the selectable calibration procedure, and the devices employed for such purpose. Section 4 describes the data employed for training the calibration method and the data used for the validation of the predictive algorithm to detect AF. Finally, Section 5 concludes.

## 2. Normal Sinus and Atrial Fibrillation Classification Scheme

Recently, we have consistently applied symbolic recurrence quantification analysis (SQRA) for the detection of atrial fibrillation [31]. SRQA provides a powerful framework to study the dynamic behavior of a system. In particular, we developed a novel algorithm based on a logistic model that we called ReAD-AF (Recurrence Analysis to Detect Atrial Fibrilation). The algorithm is intended to determine whether a patient is in a normal state (normal sinus rhythm NS) or with AF. The solution is based on the RR interval of an ECG signal as the unique input, requiring minimum pre-processing computational cost. Specifically, we constructed a logistic regression algorithm that employs SRQA over RR interval time series. Here, we summarize its operation and main features. Additionally, the pseudocode implementing the algorithm is included in Appendix B. The proposed approach uses a symbolization procedure based on neighboring permutations. That is, given an RR interval time series ${\left\{x_{t}\right\}}_{t=1}^{T}$, its space representation can be built by means of Taken’s time delay method [33], so the space vector will be ${\bar{x}}_{t}=\left(x_{t},x_{t+1},\ldots ,x_{t+m-1}\right)$ for an embedding dimension *m*. A symmetric group ($S_{m}$) of order *m*! is defined as a group that contains all the permutations of length m. In turn, each permutation is defined as a symbol π. It should be mentioned that each vectorial time series from ${\bar{x}}_{t}$ is called *m*-history. Finally, a symbolization map $S({\bar{x}}_{t})$ transforms the *m*-history of the vectorial time series into a sequence of symbols π representing ordinal patterns of length *m*, so that

(1) $${\bar{x}}_{t}\mapsto S\left({\bar{x}}_{t}\right)=\pi .$$

As an example, the transformation of the RR interval sample set (2) into a sequence of symbols (3) is shown as follows.

(2) $$x_{t}=\left(0.55,0.65,0.60,0.70,0.65,0.50\right).$$

Taking into account that if m=3, the symmetric group is formed of m!=3!=6 symbols, so

(3) $$S_{3}=\left\{(2,1,0),(2,0,1),(1,2,0),(1,0,2),(0,1,2),(0,2,1)\right\}.$$

Each m-history of length 3 corresponds to a symbol (and an assigned color) of the symmetric group $S_{3}$. Thus, for t=1, ${\bar{x}}_{1}=\left(0.55,0.65,0.60\right)$, it can be observed that $x_{t+0}=0.55<x_{t+2}=0.60<x_{t+1}=0.65$ which means that ${\bar{x}}_{1}$ corresponds to a symbol of type (0,2,1). Similarly, for t=2, ${\bar{x}}_{2}=\left(0.65,0.60,0.70\right)$, it is observed that $x_{t+1}=0.60<x_{t+0}=0.65<x_{t+2}=0.70$, so ${\bar{x}}_{2}$ corresponds to a symbol of type (1,0,2).

Now, two symbols $S({\bar{x}}_{t})$ and $S({\bar{x}}_{s})$ at different instants in time (*t* and *s*) are recurrent if and only if $S({\bar{x}}_{t})$ = $S({\bar{x}}_{s})$. So, an indicator function is defined as

(4) $$SRP_{ts}=\left\{\begin{array}{c} k\;\mathrm{if}\;S\left({\bar{x}}_{t}\right)=S\left({\bar{x}}_{s}\right)=\pi_{k},\\ 0\;\mathrm{otherwise}. \end{array}\right.$$

Therefore, the Symbolic Recurrence Plot (SRP) is defined as a matrix that contains the recurrences of all the symbols provided by the indicator function. The SRP becomes a powerful tool for analyzing the dynamic behavior of the time series and can be represented in a graph where the axes represent the time indexes of the time series. Each colored dot at coordinates (t,s) in the graph means that the m-histories ${\bar{x}}_{t}$ and ${\bar{x}}_{s}$ are recurrent to the corresponding symbol. White dots mean that the m-histories are not recurrent. This enables the display of the portion of the phase space that is being visited by the system. If a dynamic change occurs, then the colored distribution of the SRP will also change.

**Figure 1:**
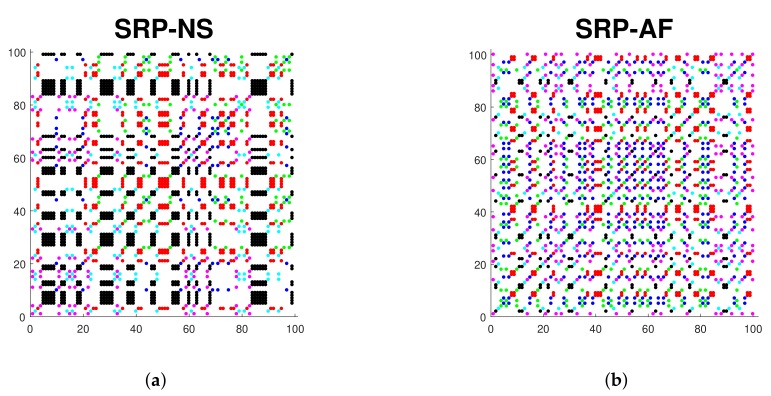
SRPs of a 102-length RR interval time series for an embedding dimension m = 3 in a normal sinus (**a**) and atrial fibrillation (**b**) patient.

Figure 1 illustrates an example of two SRPs plotted from a 102-lengh RR interval time series from a patient with normal sinus rhythm and another with atrial fibrillation. We can observe that the colored distribution is different in both graphics, which means that the dynamic behavior changes in both time series. The normal sinus rhythm SRP presents a much more structured distribution, with a remarkable predominant color (black) of symbolic recurrence. In contrast, the atrial fibrillation SRP does not exhibit a structured pattern. The symbols appear in a random way without following any particular distribution. These random patterns characterize the atrial fibrillation patient due to the non-periodic state of the RR intervals. However, the normal sinus rhythm patient presents a characteristic set of symbols that repeat periodically.

To figure out the probability of a patient being categorized as being in a NS or with AF, a logistic model is created. To estimate the model, it is necessary to define a set of covariates. These covariates are divided into two groups. The first group contains those based on the diagonal and vertical lines of the SRP, as well as the recurrence rates of each symbol. The second group is based on the distribution of the RR intervals and comprises the mean, median, Pearson coefficient of variation, and the coefficient of variation of the median. These covariates allow the discrimination between normal sinus rhythm and atrial fibrillation patients. The logistic model is generated through a receiver operating characteristic (ROC) curve analysis in order to compute a probability threshold. So, a patient with an estimated probability above this threshold is classified as having AF.

In order to build this curve, three metrics are assessed. Namely, sensitivity, specificity, and accuracy. To this end, the number of true positive (TP), true negative (TN), false negative (FN), false positive (FP), sensitivity (Se) or the true positive rate $Se=\frac{TP}{TP+FN}$, and the false positive rate FPR given by $FPR=\frac{FP}{TN+FP}$ have to be defined. In addition, specificity (Sp) is also defined as Sp=1−FPR. Finally, accuracy (ACC) is determined as $ACC=\frac{TP+TN}{TP+FN+FP+TN}$. Taking all these metrics into account, the ROC curve is constructed using the (FPR,Se) points, and the optimal threshold τ parameter is the one that minimizes the distance between the points (FPR, Se) and (0,1),

(5) $$\tau =\underset{\tau }{\arg\min}\left\{FPR_{\tau }^{2}+{\left(1-Se_{\tau )}\right)}^{2}\right\}.$$

In the original paper [31] it was shown that sensitivity, specificity and accuracy increased with the window size selected. Sensitivity always reached values above 96% and specificity slightly lower with values above 94.8% for the smallest window sizes. Finally, accuracy achieved values of 95.4% presenting good predictive power.

To validate the algorithm, a k-fold cross-validation procedure was performed. The logistic model was fitted with a training set and further evaluated with a test set. The entire data set is publicly available and provided by Physiobank [34].

The ReAD-AF algorithm was shown to be robust and capable of discriminating AF patients with high precision. Indeed, the ReAD-AF algorithm developed can work with only the RR interval feature as input, which implies a reduction of the computational cost of the model, enabling portability to mobile platforms such as smartphones.

In addition to this classification algorithm based only on RR intervals as input, we want to further contribute with a methodology to help practitioners develop an application based on algorithms that can be applied to the growing low-cost biomedical sensor field. Thus, the next section illustrates a framework for such purpose.

## 3. Prototype Application

**Figure 2:**
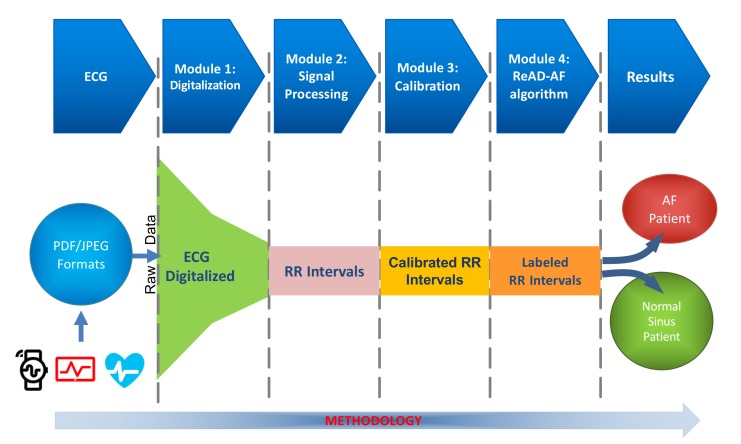
Block diagram showing the main modules that shape the prototype application developed. After the entire procedure, the objective is to determine if a patient is with normal sinus rhythm (NS) or with atrial fibrillation (AF).

In this section we look deeper into the application modules developed. A general block diagram of this application is depicted in Figure 2. A detailed description of these main modules as well as how they work is discussed below.

The first module uses an ECG signal (acquired from a low-cost device) as input in PDF or JPEG format and digitalizes it. Satisfying the well-known sampling theorem, the module takes as its main parameter double the bandwidth of the device under consideration. Selecting double the bandwidth is enough to be able to obtain all the signal information. The background color of the electrocardiogram and the duration of the ECG are also important parameters to take into account. Since direct access to data captured by a particular sensor usually follows proprietary protocols, this digitalization procedure enables the implementation of a generic application, regardless of the low-cost commercial device finally selected to provide the input ECG.

The next module is responsible for detecting the inter-beat (RR) intervals also known as heart rate variability (HRV) in an ECG. This metric measures the specific changes in time (or variability) between successive heartbeats. This time variation (usually expressed in milliseconds or seconds) is controlled by a primitive part of the nervous system called the autonomic nervous system (ANS). RR intervals are one of the indicators of a person’s state of health, fitness, and recovery. There are many algorithms available in the open literature for detecting heart rate variability (HRV). For instance, the well-known Pan and Tompkins algorithm [35] detects QRS waves, or QRS complex, based on the slope, amplitude, and width of the ECG signal. Our algorithm is divided into two stages; the pre-processing and the decision-making process. In the pre-processing phase, the signal is prepared by removing noise, smoothing the signal, and amplifying the QRS slope. Then, in the decision stage only signal peaks are obtained. It is also possible to apply a set of well-proven tools developed by MIT [36,37], enabling the detection of QRS complex and RR intervals. In our prototype, the implementation of the RR interval method is carried out with a native MATLAB function (findpeaks).

Once the RR intervals have been acquired, our application could take them directly to further feed a third module for calibrating these data obtained by a low-cost portable device against professional 12-lead gold standard electrocardiograph equipment in order to achieve more precise data and later diagnosis. This calibration is usually based on a linear regression model aimed at improving the accuracy of the data acquired by the low-cost device finally employed. The calibration will allow us to analyze the ECG signal with the highest degree of accuracy. Interestingly, we prove that measures obtained from SRQA are invariant under monotonic transformations (see Appendix C), which means that the symbolic recurrence measures employed as covariates in our algorithm are not affected in those cases in which the calibration processes are based on linear regression. That is, raw uncalibrated data can be used to feed our algorithm directly. However, for practitioners who might use different approaches, a possible calibration method will also be introduced below.

The last module, implementing the ReAD-AF scheme, is in charge of assisting the AF classification. Its power of classification is addressed in Section 4.2.

All of these modules are intended to illustrate a general methodology since, as commented above, commercial sensor applications are mainly based on proprietary protocols. That is, the data acquired is not directly accessible, and only an ECG representation, commonly in PDF or JPEG format, is available.

### 3.1. Module 1: ECG Digitization

**Figure 3:**
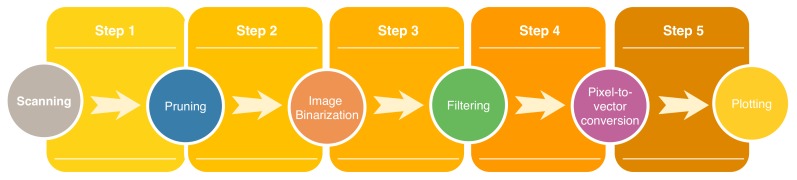
Block diagram showing the steps of the digitalization procedure.

To analyze the ECG signals comprehensively, we first require a technique that enables the digitalization of a graph in PDF or JPEG formats, which are the most common output formats of commercially available sensors. This will provide independence to the data acquisition platform and simplicity in the generalization of our methodology. For this purpose, we have customized a procedure implementing this functionality [38]. The sequence of actions for the digitization process of the ECG signal in PDF or JPEG format are shown in Figure 3, in addition to a detailed description of each one.

The first phase of our proposal is the scanning of the ECG signal. To do this, a MATLAB-scan-function (imread) is applied at a resolution of 600 dpi which takes a PDF file containing the ECG as an input parameter.

To perform the scan a portion of the PDF document must first be selected. The complete PDF document contains additional information, such as the patient’s name, or his/her date of birth, among other things. Therefore, it is necessary to prune the signal to be digitalized.

In the image binarization phase, RGB color images are converted into binary images. This process reduces the number of colors at the binary level, resulting in a clear reduction of the memory needed to store the signal. This function also simplifies the processing of the binarized image in comparison to the original image. However, ECG charts are always represented graphically in a grid, which interferes with the binarization process of the image. One way to eliminate or erase this grid in the background of the image is to set an RGB threshold. In detail, all interfering colors are enclosed in the threshold and transformed into white before starting the binarization mechanism. The end result is a white background with the ECG signal in a selectable (e.g., blue) color.

**Figure 4:**
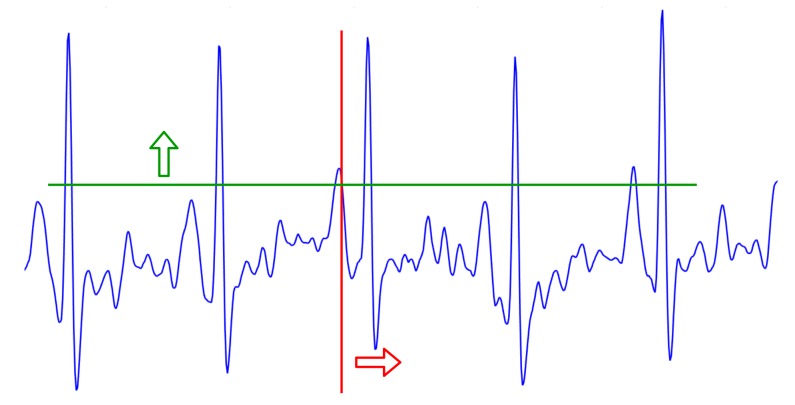
Horizontal scanning (green line) vs vertical scanning (red line). Arrows represent the direction of the scanning process.

Some noise might appear, characterized by isolated black and white pixels in the binarized image. This may impair the subsequent analysis of the data. Therefore, a filtering function has also been implemented to eliminate these error pixels. To extract the ECG signal graph from the two-dimensional binarized image, horizontal scanning is usually employed. However, our module implements vertical scanning to identify the ECG signal pixels. The vertical scanning is selected to achieve faster and more accurate digitalization. While with horizontal scanning pixel location identification requires an iterative process, with vertical scanning, each pixel directly represents a (x, y) position coordinate of the graph, which results in a one-dimensional vector. Figure 4, below, illustrates both types of scanning.

In order to offer greater data accuracy, vectors double the number of columns of pixels analyzed, because some peaks are plotted as successive vertical black lines in scenarios with low resolution images. Each component of these vectors is a complex number. The two components of the same column have an identical real part, which is the column index of the pixel matrix. The two consecutive imaginary parts (of the same real part) quantify the upper and lower limits of the vertical black line, that is, of the ECG signal under study. For example, the 2D-vector T=[…;12+27i;12+28i;…] means that the vertical black line spans from line 27 to line 28 in column 12.

The digitalization algorithm searches the data from the first pixel (bottom left) to the last one (top right). Once the original axes have been deleted from the ECG graph, the value of the bottom vertical and the first column positions are used as a reference for the ECG scanning.

To convert the above mentioned 2D vector into a one-dimensional vector, the algorithm computes the modal distance (μ) between the imaginary part of two successive components; that is, the number of vertical pixels comprising the signal in a specific column. If the difference between the imaginary components of the two coordinates is within the limit μ, then only the imaginary part of the second element is included in the 1D version of the vector. This value is assumed as a reference (λ) to the R peak identification in the next column analysis. If this difference is greater than μ, the module of the difference between the λ and each of the two components is calculated. The stored 1D value is the greatest obtained value. One can often find portions of the graph where the drawing is no longer continuous. In order to provide a consistent 1D vector, the components in these positions are estimated through linear interpolation. Finally, the digital signal is plotted taking into account this final one dimensional vector and the sampling frequency.

For a better understanding of the digitalization process, the pseudocode of the employed methods is also shown below in Algorithm 1, as well as an explanation of the sequence of actions. The input is an ECG in PDF or JPEG format. Then, step 2 allows the reading of the input file. Step 3 applies an interactive image pruning operation to the image previously read. After this, the original image is transformed into a binary one. This is denoted in step 4, during which a binary mask and a composite image are generated. The composite image shows the original RGB pixel values under the mask. Step 5 indicates that portions with fewer than P pixels, in our case 1260 pixels, are removed from the binary image, resulting in a clearer signal where all the pixels but the ECG signal curve are eliminated. Then, a shrink function is applied in step 6 for a better union between points. Finally, vertical scanning is implemented in step 7 for the remaining steps to convert the binarized image into a 1D vector, and the resulting output signal is then plotted in MATLAB format (.fig).
**Algorithm 1** PDF to MATLAB1:**Input: PDF file (.pdf)**2:r← read(PDF)3:p← prune(*r*)4:b← createBinaryMask(*p*)5:bw← bwAreaOpen(b,1260)6:m← bwMorph(bw,shrink,2.5)7:vector← pixelToVector(m)8:**Output: MATLAB signal (.fig)**

**Figure 5:**
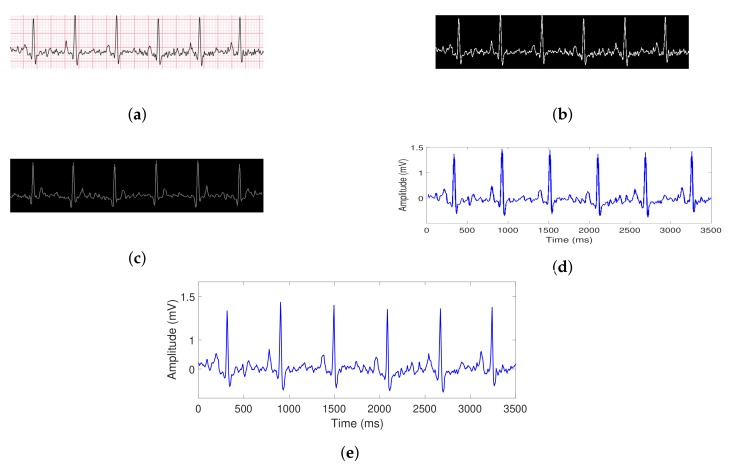
Transformation process of the signal from PDF or JPEG formats (**a**), then a binary mask image is obtained (**b**) and after that, some pixels are removed (**c**). Finally, a shrink operation is applied to capture only useful information (**d**), and then the output digitalized signal (**e**) is shown.

Figure 5 shows the digitalization procedure step by step. At each step, the image is carried out by a transformation to be able to obtain the final digitalized signal.

### 3.2. Module 2: Signal Processing and Filtering

Once the digital output signal is obtained, the processing phase to attain the RR intervals starts. First, in order to smooth the data obtained from Module 1 in Section 3.1 we apply the MATLAB function filter(b,a,x) that uses the rational transfer function

(6) $$Y\left(z\right)=\frac{b\left(1\right)+b\left(2\right)z^{-1}+\cdots +b\left(n_{b}+1\right)z^{-n_{b}}}{a\left(1\right)+a\left(2\right)z^{-1}+\cdots +a\left(n_{a}+1\right)z^{-n_{a}}}X\left(z\right),$$

with a=1 and b=[0.20.20.20.20.2] providing a filter of fourth order.

**Figure 6:**
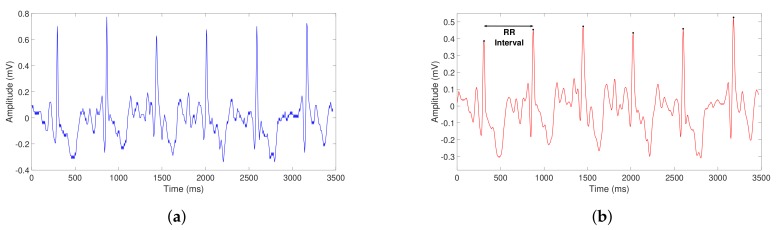
Transformation of the signal before (**a**) and after (**b**) applying the filter function

As an example, the functionality of the filter is illustrated in Figure 6.

The next stage of the analysis of the ECG signal is peak detection. To do this, the findpeaks function of MATLAB is employed, adjusting some parameters of this function according to the type of signal under consideration. This function returns a vector with the local maxima (peaks) of the input signal vector (data), together with the position index in which the peak is located.

The resulting vector allows the computation of RR intervals in ECG signals (see Figure 6, red signal); which, as previously mentioned, are essential values to provide as input to our classification algorithm.

### 3.3. Module 3: Calibration Procedure

In order to exemplify the calibration procedure, we employ a low-cost, portable, commercial device called Kardia Mobile (AliveCor Inc., San Francisco, CA, USA). It enables one-lead ECG recording. Note that any other low-cost alternative device could be used, provided that it offers an output ECG in PDF or JPEG format, as is commonly the case. Kardia by AliveCor is one of a family of mobile, clinical-quality electrocardiogram (ECG) recorders.

**Figure 7:**
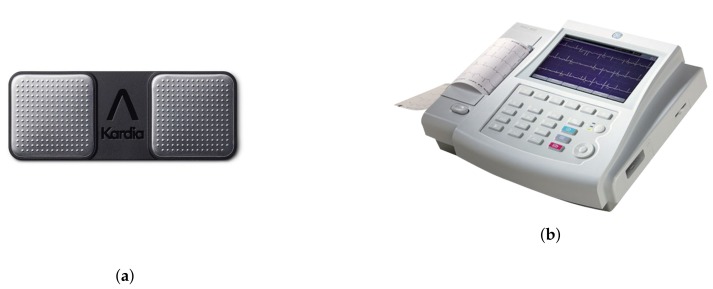
Kardia Mobile device by AliveCorc (**a**) and MAC800 by General Electric Healthcare (**b**); both employed for the calibration procedure.

Furthermore, we employ professional 12-lead gold standard ECG ground truth equipment, generally used in many hospitals to monitor patients. In particular, the MAC800 by General Electric Healthcare is the one selected. The MAC 800 system provides the ability to store, analyze and display ECGs in a huge variety of formats with utmost accuracy. Both devices are shown in Figure 7.

Kardia Mobile and MAC800 are capable of monitoring patients and representing their electrocardiograms. However, the 12-lead MAC800 is a much more accurate device than the single lead Kardia Mobile. Therefore, to be comparable, data gathered by Kardia Mobile have to be calibrated, taking as a reference the data acquired by the MAC800 gold standard in order to attain the same quality results.

**Figure 8:**
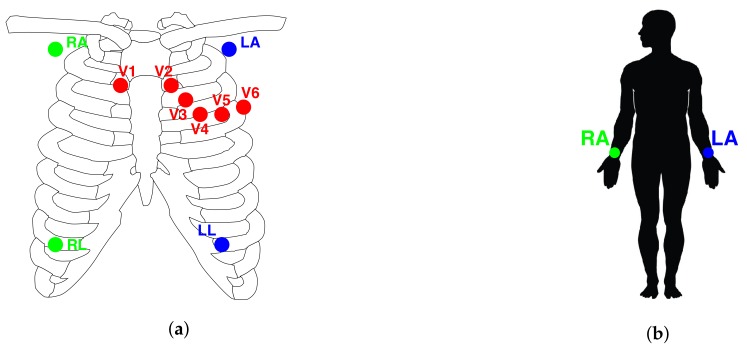
Electrocardiograph (ECG) placement configuration in both devices MAC800 (**a**) and Kardia Mobile (**b**)

**Figure 9:**
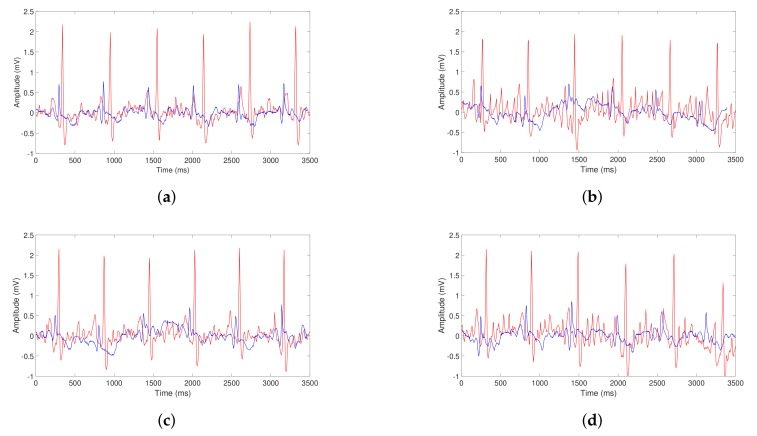
Different ECG deviations from (**a**–**d**) of the Kardia Mobile (blue) with respect to the MAC800 (red) equipment.

To achieve comparable results, we have to adjust the data acquired by the low-cost device with respect to the data measured by the more powerful ECG equipment (gold standard). The calibration procedure is carried out by comparing the morphological signals in both devices, which is the signal contained in the Lead I of each ECG. The lead placement configuration in both devices is shown in Figure 8. Due to the fact that Kardia Mobile only has a Lead I configuration, which goes from the RA (right arm) to the LA (left arm), the lead placement of the MAC800 needs to have a Lead I as well. In our case, the Mason-Likar placement [39] is employed in MAC800 because this configuration contains the Lead I signal (other leads are not used), necessary to be compared with the Lead I signal of the Kardia Mobile. The wires and adhesive electrodes of the MAC800 are much less vulnerable to artifacts and variability, providing much cleaner and more stable ECG recording with respect to the Kardia Mobile ECG (see Figure 8). In fact, we have observed in our study that the PQ and QT intervals of the Kardia Mobile had different lengths compared to the same intervals of the MAC800 gold standard (see Figure 9). This means that the morphology of the Kardia Mobile ECG waveform slightly differs from the MAC800 signal. Consequently, it could be expected that the deviation in the waveform will affect the precision of Kardia Mobile’s RR interval data measurements, and therefore, the atrial fibrillation detection procedure.

For a deeper insight into the accuracy of the Kardia Mobile in comparison to the MAC800 equipment, a set of ECGs is taken for the same patient using both devices (Kardia Mobile and MAC800) simultaneously.

Although the absolute error of the Kardia Mobile acquired data is relatively low compared to the MAC800 recordings, it is not negligible, and the corresponding deviation should be taken into consideration. Figure 9 shows four examples of each Lead-I ECG representing the difference between the Kardia Mobile signal in blue and the MAC800 Healthcare signal in red. It should be noted that the scale in the y-axis is notably different between both devices, due to the higher gain of the MAC800 with respect to the Kardia Mobile. Note, however, that this particularly significant gap does not affect the calibration procedure since the RR interval is the metric of interest.

In order to provide a better estimation in the calibration procedure for the Kardia Mobile measurements of RR intervals -namely $RR_{KM}$- we propose a common modification of $RR_{KM}$ that reduces the deviations of the measurements given by MAC800 ($RR_{M}$) by means of a linear regression:

(7) $$RR_{M}=a+bRR_{KM}.$$

The estimated values for the linear regression parameters in Equation (7) are a=−0.011794 and b=1.0679, with a coefficient of determination of $R^{2}=0.91$, providing an excellent goodness of fit measure. Therefore, according to these estimated values, to calibrate the RR interval samples given by Kardia Mobile, we have to increase each measurement $RR_{KM}$ by 6.79% and then use a global correction of all the samples of −0.011794.

**Figure 10:**
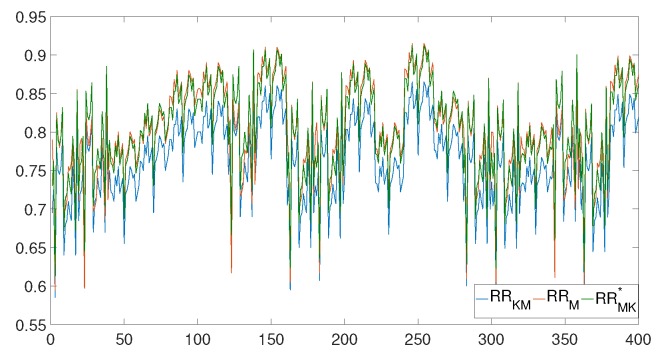
RR interval data for 20 samples from 20 different patients measured with Kardia Mobile (blue), MAC800 (red) and corrected Kardia Mobile (green).

Figure 10 illustrates how the proposed adjustment reduces the deviation of the Kardia Mobile RR intervals with respect to the RR intervals of the MAC800. Although the model has been trained with data sets of 20 patients (400 RR interval data samples), the calibration is intended to be applied to each patient individually. Thus, if we measure the RR intervals of an unseen patient with the Kardia Mobile, it will be transformed into other more precise RR intervals after applying the calibration procedure.

It is important to emphasize that we have described the calibration procedure (and its validation below) for illustrative purposes for future practitioners. The procedure is intended to improve the accuracy of the RR intervals extracted from any low-cost biomedical sensor. Nevertheless, our specific mathematical framework (ReAD-AF) is slightly affected by linear transformations and the difference before and after the calibration can be calculated (see Appendix C). However, this difference only affects the predictive power (accuracy) of the algorithm in the fourth decimal at most, as can be seen in Table 1.

## 4. Results

### 4.1. Validation of the Calibration Procedure

In order to validate the calibration procedure, we have measured the RR intervals of 20 different patients (60% men, mean age 25.3±6.2 years) with both the Kardia Mobile and the MAC800 devices, gathering 20 samples from each patient, with a total sample size of 400. Each patient was asked to provide formal consent for the ECG data acquisition. All subjects agreed to be involved in the study. The clinical state of the subjects was stable at the time of ECG acquisition. The center in which the tests were taken is the Technical University of Cartagena, whose informed consent form was approved by the local bioethics committee. After analyzing the ECGs, all subjects were with normal sinus rhythms at the time of Kardia Mobile and MAC800 ECG data acquisition. There were no patients with sustained arrhythmia. Thus, we trained a linear regression model with the RR intervals acquired from the MAC800 gold standard to correct the RR intervals provided by Kardia Mobile.

As an estimation of the error after calibration, the mean squared error (MSE) metric is employed. Specifically, given a sample ${\left\{\left(X_{i},Y_{i}\right)\right\}}_{i=1}^{n}$ of size *n* of the bidimensional variable (X,Y), the MSE measures the mean squared differences between the two variables; that is:

(8) $$MSE\left(X,Y\right)=\frac{1}{n}\sum_{i=1}^{n}{\left(X_{i}-Y_{i}\right)}^{2}$$

The MSE metric ranges from zero to infinite, especially penalizing those data points that are far from each other. In our case, the RR intervals of both the Kardia Mobile and MAC800 devices are compared by using the MSE. First, the mean squared error of the RR interval samples from 20 patients between the Kardia Mobile (without calibration, $RR_{KM}$) and the MAC800 ($RR_{M}$) device is calculated, achieving the following result (9):

(9) $$MSE\left(RR_{KM},RR_{M}\right)=\frac{1}{n}\sum_{i=1}^{n}{\left(RR_{KMi}-RR_{Mi}\right)}^{2}=0.001971.$$

Second, the mean squared error of the RR interval samples from the 20 patients between the calibrated Kardia Mobile ($RR_{KM}^{*}$) and the MAC800 device is also computed, obtaining the following result (10):

(10) $$MSE\left(RR_{KM}^{*},RR_{M}\right)=\frac{1}{n}\sum_{i=1}^{n}{\left(RR_{KMi}^{*}-RR_{Mi}\right)}^{2}=0.000355.$$

Observing these results, it is concluded that the MSE of the calibrated device is much lower. This means that when the calibration process is applied, the resulting values are closer to the values of the RR interval samples taken by the MAC800 gold standard professional equipment.

### 4.2. Validation of the Classification Model

For the atrial fibrillation detection procedure, our ReAD-AF algorithm was trained with publicly available ECGs taken from Physionet Challenge 2017 [40]. As a description of the challenge database, the training set contains single lead ECG recordings lasting from 9 s to just over 60 s, and the test set contains 3658 ECG recordings of similar duration. There are four different classes of ECG waveforms—normal rhythm, AF rhythm, other rhythm, and noisy recordings. We have selected the ECGs classified as AF and NS since the other signals are not of interest in this study. It should be noted that these reference ECG data were actually transferred from the Kardia Mobile device company to the Physionet Challenge 2017, which, without any loss of generality, was one of the reasons for selecting Kardia Mobile as the low-cost device to use in this study. Furthermore, the whole set of ECGs were calibrated through the calibration procedure mentioned in Section 3.3. Thus, we tested our algorithm with both sets of ECGs (raw and calibrated). To provide validation of the classification model, three metrics are employed. These metrics are sensitivity, specificity, and accuracy, as defined in Section 2. *Sensitivity* is a measure of the proportion of patients with AF correctly diagnosed or identified. *Specificity* refers to the proportion of healthy patients that are appropriately identified. Finally, *accuracy* measures the share of data taken from NS or AF patients that is correctly identified. The final performance attained by our algorithm is presented in Table 1.

As discussed in the previous section, and due to the fact that SRQA is invariant under monotonic transformations, Table 1 shows that the sensitivity, specificity, and accuracy attained by our logistic model in both, raw and calibrated data sets are quite similar, which means that our approach has good power performance even with non-calibrated data. This invariance property of the SRQA approach enables the obtaining of robust results with commercial low-cost sensors, because their inherent low-accuracy does not affect the final NS or AF classification.

In order to compare the results attained by our algorithm with the existing methods in literature, Table 2 shows a comparison of the sensitivity, specificity, and accuracy of some relevant algorithms.

Table 2 illustrates that our algorithm employs a relatively small number of features, which translates into a more efficient computational cost of the method. Note, however, that even using a smaller number of features, it attains good performance power in terms of sensitivity, sensibility, and accuracy; even better (in some cases) than other algorithms. More concretely, the ReAD-AF algorithm detects AF in 87.65% of the cases, discerns healthy patients in 91.86% of the cases, and its predictive power (accuracy) is 91.33%.

## 5. Conclusions

In this paper, we have described the methodology for designing a prototype, but fully functional application, which meets the following requirements: (i) it is robust against low-quality data, therefore enabling the use of multiple low-cost wrist-worn sensors generally characterized by low accuracy in comparison to professional electrocardiographs; and (ii) it is effective for analyzing dynamic changes based on a time series of RR intervals.

Symbolic recurrence quantification analysis (SRQA) is applied to implement a predictive scheme for the detection of AF by using, in principle, any commercially available sensor. Our approach only employs the RR intervals taken from an ECG signal in PDF or JPEG format, which further reduces the complexity of the pre-processing phase, enabling an efficient algorithm. Despite its good power performance for AF detection, it does not take into account the medical history of the patient and is restricted to the detection of AF. As a future research line we plan to extend the logistic model to a multinomial scenario where several types of arrhythmia (the most prevalent ones) will be classified and explained with new covariates extracted from the medical history of the patient.

In addition, it should be mentioned that our first preliminary tests show the computational efficiency of the entire application here introduced, which is a fundamental step towards its portability to mobile devices and, thus, its wide potential to be provisioned in those communities without sufficient resources to access medical care.

Finally, our study is intended to offer practitioners a foundation for further development of apps in the emerging digital health field based on low-cost sensors.

## Figures and Tables

**Table 1:** Sensitivity, specificity, and accuracy of our classification model applied to the raw data set (left) and calibrated data set (right).

Sensitivity	Specificity	Accuracy	Sensitivity	Specificity	Accuracy
0.8765	0.9186	0.9133	0.8765	0.9184	0.9131

**Table 2:** Comparison of the sensitivity, specificity, and accuracy between some Physionet Challenge 2017 methods and our algorithm.

Reference	Number of Features	Classification Method	Se	Sp	Acc
Billeci et al. [16]	30	Least Square-Support Vector Machine	0.83	0.96	0.95
Behar et al. [41]	42	Feature-Based Machine Learning	0.81	0.91	0.90
Christov et al. [42]	>40	Multi-Parametric Analysis	0.82	0.90	0.89
ReAD-AF [31]	9	Symbolic Recurrence Analysis	0.87	0.91	0.91

**Figure A1:**
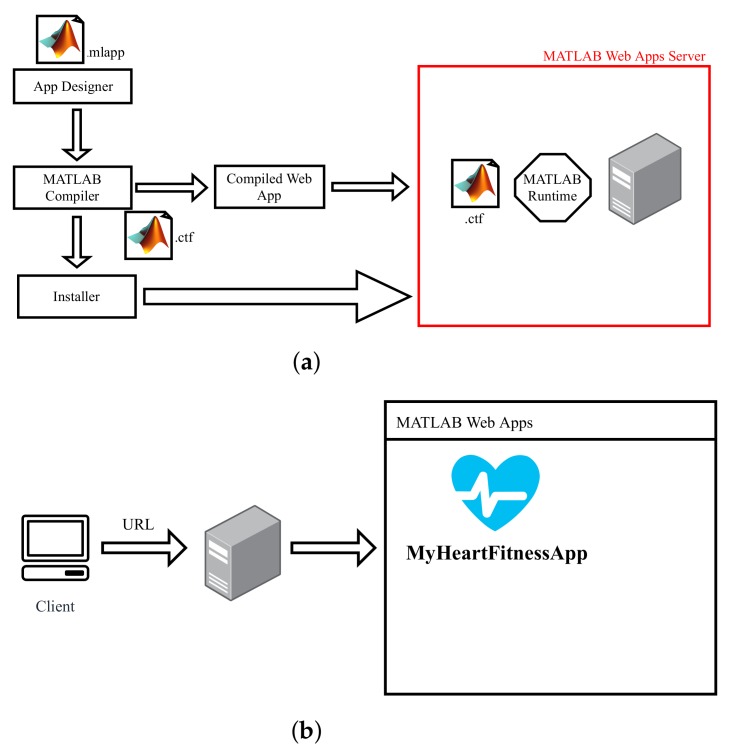
Functional diagram of the application implemented in MATLAB (**a**). Remote access to the developed application in the cloud (**b**).

## Appendix B

**Algorithm A1** ReAD-AF
- 1: **Input: RR interval data set of a patient** - 2: raw_data← load(RR) - 3: data← splitRR(raw_data, w) ▹ Split RR intervals in windows of size w - 4: meanRR← mean(data) ▹ Mean - 5: medianRR← median(data) ▹ Median - 6: VRR← std(data)/mean(data) ▹ Pearson coefficient of variation - 7: VmeRR← sum(abs(data-medianRR))/medianRR; ▹ Mean absolute dispersion - 8: **SRR**: symbolic recurrence rate - 9: **SRP**: symbolic recurrence plots - 10: **Vinc(1,3)**: Shannon entropy prob. distrib. vertical line lengths; SRP for increasing symbol {i} - 11: **Vinc(1,4)**: Mean length of vertical lines; SRP for increasing symbol {i} - 12: **Vdec(1,3)**: Shannon entropy prob. distrib. vertical line lengths; SRP for decreasing symbol {i} - 13: **Vdec(1,4)**: Mean length of vertical lines; SRP for decreasing symbol {i} - 14: **DETt**: The percentage of recurrence points which form diagonal lines; SRP for all symbols - 15: **ENTRt**: Shannon entropy of the probability distribution of the diagonal; SRP for all symbols - 16: Matrix← func_SRP(data, m) ▹ Matrix SRR for all symbols - 17: [Vinc(1,3),Vinc(1,4)]← recu_SRQA(Matrix {i}) - 18: [Vdec(1,3),Vdec(1,4)]← recu_SRQA(Matrix {i}) - 19: [DETt,ENTRt]← recu_SRQA(Matrix) - 20: **measure**: Storage of all metrics in a matrix - 21: measure←[meanRR,medianRR,VRR,VmeRR,DETt, ENTRt,Vinc(1,3),Vdec(1,3),Vinc(1,4),Vdec(1,4)] - 22: **pos_af**: The boolean vector identifying the AF windows - 23: mod← fitglm(measure,’Distribution’,’binomial’,pos_af) ▹ Compute the logistic model - 24: prob← mod.Fitted.Probability ▹ Compute the estimated probability of AF - 25: Tlist←[0.001:0.001:1] - 26: **for** j=1 **to** j=lenght(Tlist) **do** - 27: ths← Tlist(j) ▹ Compute the threshold - 28: TP← length(find(prob(find(pos_af>0))>ths)) ▹ True Positive - 29: TN← length(find(prob(find(pos_af==0))<ths)) ▹ True Negative - 30: FN← length(find(prob(find(pos_af>0))<ths)) ▹ False Negative - 31: FP← length(find(prob(find(pos_af==0))>ths)) ▹ False Positive - 32: TPR← TP/(TP+FN) ▹ Sensitivity or True Positive Rate - 33: FPR← FP/(FP+TN) ▹ False Positive Rate - 34: SPC← 1-FPR ▹ Specificity - 35: ACC← (TP+TN)/(TP + FN + FP + TN) ▹ Accuracy - 36: **end for** - 37: τ: Optimal threshold parameter - 38: $\tau \leftarrow min\{FPR_{\tau }^{2}+{\left(1-Se_{\tau }\right)}^{2}\}$ ▹ Compute the optimal threshold for AF classification - 39: **if** prob≥τ **do** - 40: patient classified as AF - 41: **else** - 42: patient classified as NS - 43: **end if** - 44: **Output: Classified patient**

## Appendix C

Let ${\left\{x_{t}\right\}}_{t\in I}$ be a real valued time series and f:R→R a monotonic real map transforming $x_{t}\to f\left(x_{t}\right)=y_{t}$. Since *f* is a monotonic map, it preserves the order relations ″<″ and ″>″, that is if $x_{t}<x_{t+k}$ (respectively $x_{t}>x_{t+k}$) then $f\left(x_{t}\right)=y_{t}<f\left(x_{t+k}\right)=y_{t+k}$ (respectively $f\left(x_{t}\right)=y_{t}>f\left(x_{t+k}\right)=y_{t+k}$). Therefore, $S\left({\bar{x}}_{t}\right)=S\left({\bar{y}}_{t}\right)$ for all t∈I and thus, the symbolic recurrence measures associated to the time series ${\left\{x_{t}\right\}}_{t\in I}$ and ${\left\{f\left(x_{t}\right)=y_{t}\right\}}_{t\in I}$ are the same.

On the other hand, the covariates used in the logistic model for AF classification based on RR interval measures given in [31] are divided into two groups. The first group is formed of covariates coming from symbolic recurrence measures. The second group is formed of the arithmetic mean ($\bar{RR}$), median ($Me_{RR}$), Pearson coefficient of variation ($V_{RR}=\frac{s_{RR}}{\bar{RR}}$ where $s_{RR}$ is the standard deviation of RR) and coeficient of variation of the median ($V_{Me}=\frac{D_{Me_{RR}}}{Me}$ where $D_{Me_{RR}}$ is the mean absolute deviation of RR with respect to $Me_{RR}$).

Let the log-odds of the logistic model be

(A1) $$log\left(\frac{p}{1-p}\right)=\alpha_{0}+\alpha_{1}\bar{RR}+\alpha_{2}Me_{RR}+\alpha_{3}V+\alpha_{4}V_{Me}+\sum_{i}\beta_{i}srm_{i}$$

where $srm_{i}$ are symbolic recurrence measures. Denote by $p_{KM}$ and $p_{M}$ the probabilities of AF for the Kardia Mobile measurements of RR interval and its adjustment given by a linear transformation, namely $RR_{KM}$ and $RR_{M}=a+bRR_{KM}$, respectively. It is straightforward to check that ${\bar{RR}}_{M}=a+b{\bar{RR}}_{KM}$, $Me_{RR_{M}}=a+bMe_{RR_{KM}}$, $s_{RR_{M}}=\left|b\right|s_{RR_{KM}}$, and $D_{Me_{RR_{M}}}=bD_{Me_{RR_{KM}}}$. Then, since the symbolic recurrence measures are invariant under monotonic transformations, it follows that the difference of the log-odds for $RR_{M}$ and $RR_{KM}$ is given by:

(A2) $$\begin{array}{cc} log\left(\frac{p_{M}}{1-p_{M}}\right)-log\left(\frac{p_{KM}}{1-p_{KM}}\right)= & a\left(\alpha_{1}+\alpha_{2}\right)+\left(b-1\right)\alpha_{1}{\bar{RR}}_{KM}+\left(b-1\right)\alpha_{2}Me_{KM}\\ & -\frac{\alpha_{3}a}{a+b{\bar{RR}}_{KM}}V_{RR_{KM}}-\frac{\alpha_{4}a}{a+bMe_{KM}}V_{Me_{RR_{KM}}}. \end{array}$$

Therefore, when *a* is close to 0 and *b* is close to 1, as is the case in our adjustment (a=−0.011794 and b=1.0679), the difference of the log-odds computed in (A2) is very small, which means that the predicted power of the logistic model is not affected by the proposed linear adjustment.
